# Pharmaceutical policies in Australia and New Zealand

**DOI:** 10.1186/2052-3211-8-S1-K5

**Published:** 2015-10-05

**Authors:** Zaheer-Ud-Din Babar

**Affiliations:** 1 School of Pharmacy, Faculty of Medical and Health Sciences, University of Auckland, Private Mail Bag 92019, Auckland, New Zealand

## 

This presentation will cover the pharmacy systems in Australia and New Zealand, the funding and reimbursement of medicines, the medicines supply system as well as an overview of medicine pricing policies in both countries.

New Zealand has a very effective monopsony purchaser, the Pharmaceutical Management Agency of New Zealand (PHARMAC). PHARMAC negotiates the prices of inpatient, outpatient medicines, vaccines and medical devices, and manages a capped national budget for outpatient and cancer pharmaceuticals. PHARMAC uses a variety of mechanisms to obtain lower prices, including competitive tendering, sole supply contracts, reference pricing, bundling deals, risk sharing agreements and promoting use of generics. As a result, New Zealanders have universal and nationally consistent pharmaceutical coverage, with lower patient pharmaceutical co-payments than many comparable countries.

In Australia, the Pharmaceutical Benefits Scheme (PBS) is the programme that provides subsidised prescription medicines to all Australians. In Australia several pricing reforms have been done, including a recent one in 2014. Australia also uses reference pricing for generic medicines and for groups of medicines with similar safety and health outcomes that can be used interchangeably. Overall, these policies have been effective in decreasing medicines prices and pharmaceutical expenditure. However, the critics argue that the Australia has higher prices of generic medicines when compared with New Zealand and the United Kingdom.

Australia and New Zealand both have excellent national medicines policies, which ensure the equitable access to cost-effective and safe medicines. In both countries, pharmacoeconomic analysis is compulsory to select medicines for reimbursement. New Zealand is able to achieve savings because of a combination of program budgeting and price negotiations; however, New Zealand has also been criticised because fewer medicines are available in New Zealand as compared with Australia. However, there is a dearth of research on whether or not the lack of access to some innovative medicines in New Zealand, or switching patients to different brands of medicines, adversely affects patient outcomes.

The topical issues for both countries in medicines policies include trade negotiations with the USA, access and funding of cancer medicines and the ageing population in both countries.

